# Machine Learning of All Mycobacterium tuberculosis H37Rv RNA-seq Data Reveals a Structured Interplay between Metabolism, Stress Response, and Infection

**DOI:** 10.1128/msphere.00033-22

**Published:** 2022-03-21

**Authors:** Reo Yoo, Kevin Rychel, Saugat Poudel, Tahani Al-bulushi, Yuan Yuan, Siddharth Chauhan, Cameron Lamoureux, Bernhard O. Palsson, Anand Sastry

**Affiliations:** a Department of Bioengineering, University of California San Diego, La Jolla, California, USA; b Novo Nordisk Foundation Center for Biosustainability, Technical University of Denmark, Lyngby, Denmark; U.S. Department of Energy Joint Genome Institute

**Keywords:** Mycobacterium tuberculosis, gene regulation, independent component analysis, machine learning, transcriptomics

## Abstract

Mycobacterium tuberculosis is one of the most consequential human bacterial pathogens, posing a serious challenge to 21st century medicine. A key feature of its pathogenicity is its ability to adapt its transcriptional response to environmental stresses through its transcriptional regulatory network (TRN). While many studies have sought to characterize specific portions of the M. tuberculosis TRN, and some studies have performed system-level analysis, few have been able to provide a network-based model of the TRN that also provides the relative shifts in transcriptional regulator activity triggered by changing environments. Here, we compiled a compendium of nearly 650 publicly available, high quality M. tuberculosis RNA-sequencing data sets and applied an unsupervised machine learning method to obtain a quantitative, top-down TRN. It consists of 80 independently modulated gene sets known as “iModulons,” 41 of which correspond to known regulons. These iModulons explain 61% of the variance in the organism’s transcriptional response. We show that iModulons (i) reveal the function of poorly characterized regulons, (ii) describe the transcriptional shifts that occur during environmental changes such as shifting carbon sources, oxidative stress, and infection events, and (iii) identify intrinsic clusters of regulons that link several important metabolic systems, including lipid, cholesterol, and sulfur metabolism. This transcriptome-wide analysis of the M. tuberculosis TRN informs future research on effective ways to study and manipulate its transcriptional regulation and presents a knowledge-enhanced database of all published high-quality RNA-seq data for this organism to date.

**IMPORTANCE** Mycobacterium tuberculosis H37Rv is one of the world's most impactful pathogens, and a large part of the success of the organism relies on the differential expression of its genes to adapt to its environment. The expression of the organism's genes is driven primarily by its transcriptional regulatory network, and most research on the TRN focuses on identifying and quantifying clusters of coregulated genes known as regulons. While previous studies have relied on molecular measurements, in the manuscript we utilized an alternative technique that performs machine learning to a large data set of transcriptomic data. This approach is less reliant on hypotheses about the role of specific regulatory systems and allows for the discovery of new biological findings for already collected data. A better understanding of the structure of the M. tuberculosis TRN will have important implications in the design of improved therapeutic approaches.

## INTRODUCTION

Mycobacterium tuberculosis is the second leading cause of death from a single infectious agent (the first being COVID-19) and one of the top 10 causes of death worldwide (1). The evolutionary success of M. tuberculosis is, in part, due to its adaptability to various environments, which is largely driven by its transcriptional regulatory network (TRN) (2–4). The TRN coordinates the expression of genes across various environmental conditions such as hypoxia, starvation, oxidative stress, and infection events. Given the global health impact of the pathogen, a deep understanding of its TRN is of fundamental importance.

Previous efforts to elucidate the TRN have typically consisted of characterization of individual transcription factors (TFs) using transcriptional profiling of TF knockout and overexpression strains, chromatin immunoprecipitation (ChIP), and similar methods. These efforts are extremely important for gaining mechanistic understanding and providing gold standard regulon annotations, but they are time-consuming, expensive, and often not predictive of transcriptomic data (5, 6). Global characterization of the TRN based on ChIP and TF overexpression has yielded consensus motifs for many TFs, as well as interesting observations about the widespread binding of TFs with fairly limited active regulation (7). Another global study used clustering of gene expression levels and motif analysis to enumerate a genome-scale TRN (8). These works serve as a strong foundation for understanding gene expression regulation in M. tuberculosis, but new approaches which take advantage of the large amounts of new data available and more directly quantify TF activities are needed.

One approach to TRN elucidation, which has been successfully applied to other microorganisms, is the decomposition of compendia of RNA-sequencing (RNA-seq) data using independent component analysis (ICA) (9–11). This approach identifies independently modulated gene sets (iModulons) by decomposing an initial gene expression compendium **X** into two new matrices: **M**, which links genes to iModulons and quantifies the strength of a regulator’s effect on a gene’s expression level, and **A**, which links iModulons to samples and quantifies the amount of regulator activity under each condition. In one study of over 40 TRN inference methods, ICA was the best at recovering known signals (12). Unlike regulons, which are defined from the bottom up using biomolecular data, iModulons are driven purely from the top down by statistical decomposition of transcriptomic data. ICA has been performed on transcriptomic data compendia for E. coli, S. aureus, B. subtilis, and S. acidocaldarius, and has facilitated interpretation of complex TRN responses and the discovery of new transcription factors (9–11). While global statistical analysis of expression data to identify transcriptional regulators has been performed on M. tuberculosis (7, 8), iModulon analysis can provide a novel perspective because (i) it directly infers TF activity levels, which significantly reduces the dimensionality of differential expression analysis, (ii) genes can be involved in more than one iModulon, which quantitatively captures coregulation more accurately, and (iii) it can easily be scaled to utilize the vast amount of newly available data (7, 8).

It should be noted that iModulon analysis has some key limitations. The first is that iModulons require nonnormal distributions across data sets, and thus a lack of data or insufficient regulator activation can prevent the algorithm from identifying key regulons. This is also why we require a large diversity of conditions to obtain a useful TRN from ICA (13). Second, in order to address batch effects, each project within the data set must be centered to a baseline condition within that project, which makes activity comparisons between projects complicated (14). Additionally, while the unsupervised nature of ICA is an unbiased approach, gene thresholding and enrichment annotations rely on existing TRN annotations. We seek to address these limitations by using as much available data as possible, drawing conclusions about activity levels within projects only, and carefully comparing each iModulon’s membership to known regulons in search of gaps in the existing annotations.

In order to gain deeper insight into the structure and operation of M. tuberculosis*’* TRN, we performed ICA decomposition using all publicly available RNA-seq data. We compiled 657 high quality RNA-seq expression profiles from NCBI Sequence Read Archive (15) and extracted 80 robust iModulons using our rigorous pipeline (14). We then utilized iModulons to interpret transcriptional responses and discover molecular actors in M. tuberculosis transcriptional regulation by: (i) quantitatively describing the organization of the TRN, 2) elucidating the function of new transcription factors, 3) defining transcriptional shifts that occur across changes in carbon sources, oxygen levels, and infection states, and 4) using iModulon clustering to identify a core stress response stimulon. All the work described in this paper can be found at iModulonDB.org, an interactive portal for researchers to explore interactive iModulon dashboards and download the data used in this study. In addition, we have provided an open-source platform for researchers to infer iModulon activities for any new transcriptomic data sets at https://github.com/Reosu/modulome_mtb.

## RESULTS

### Independent component analysis of publicly available data reveals 80 transcriptional modules for M. tuberculosis.

**FIG 1:**
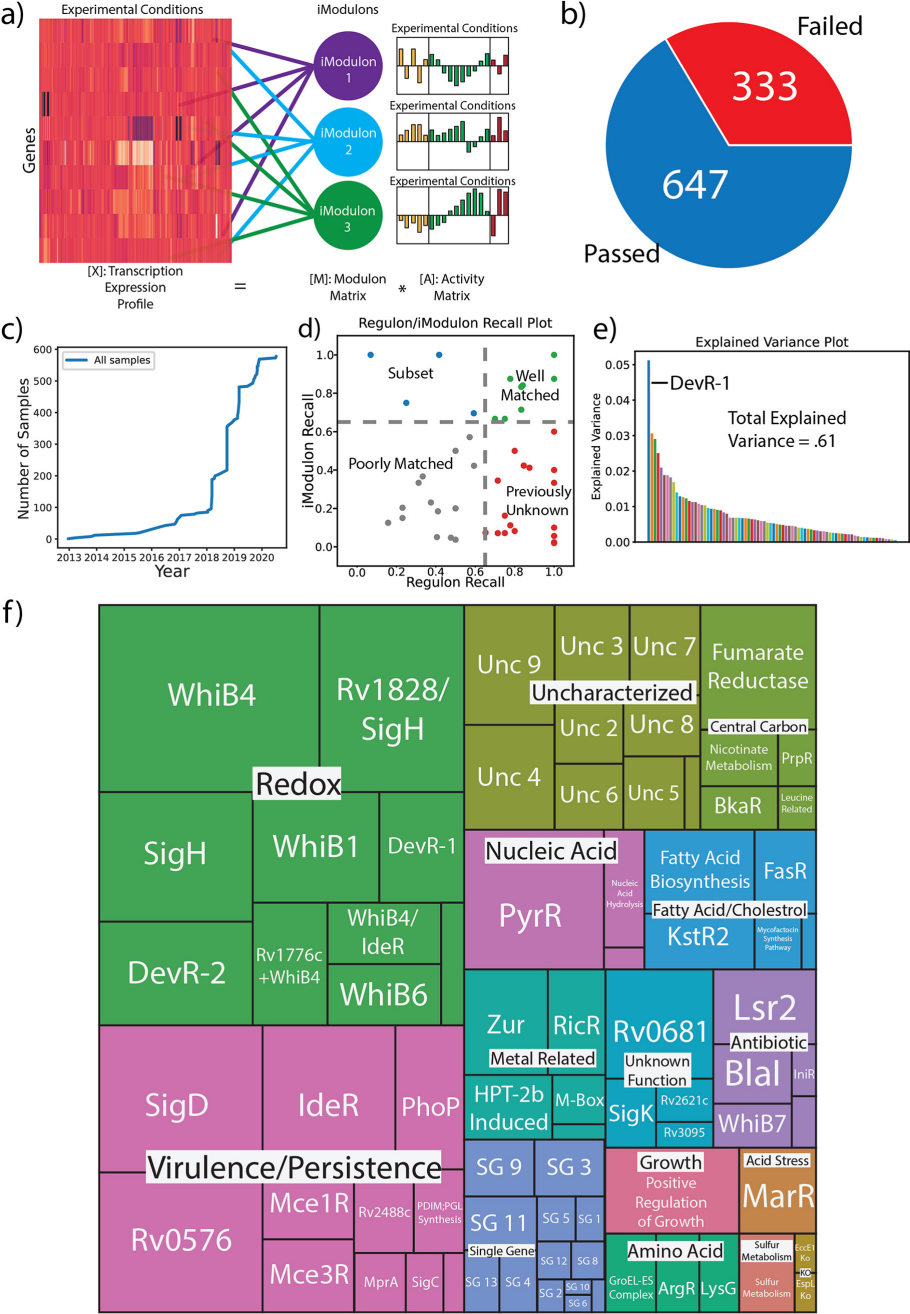
QC/QA, ICA Decomposition, and iModulon Characterization of M. tuberculosis RNA-seq Data from Sequence Read Archive. (A) iModulons are clusters of genes computed by decomposing RNA-Seq data into independently modulated sets (9). (B) Percentage of samples with metadata that passed and failed the QC/QA process. The RNA-seq data and associated metadata from 980 H37Rv SRA samples were processed, and 647 samples passed all QC/QA metrics. (C) A timeline of the number of high quality samples (samples that passed QC/QA) used in this study added to the Sequence Read Archive. (D) Scatterplot comparing the Regulon Recall to the iModulon Recall. iModulon Recall is defined as the number of shared genes divided by all genes in the iModulon, while Regulon Recall is defined as the number of shared genes divided by all the genes found in the regulon. iModulons in green are considered well matched, those in red contain mostly uncharacterized genes, those in blue are considered to be subsets of the regulon (i.e regulons can have multiple iModulons showing the dynamic dimensionality of the regulon), and those in gray only have a slight match. (E) Plot detailing how much explained variance is captured by each iModulon. Most iModulons capture relatively small amounts of explained variance, with the DevR-1 capturing the most variance in M. tuberculosis. (F) A treemap that organizes the iModulons by category. The size of each iModulon box corresponds with how many genes were found within that iModulon.

In order to capture the spectrum of M. tuberculosis*’s* transcriptional response, we scraped all publicly available transcriptomic data found in NCBI’s Sequence Read Archive (SRA) and obtained 980 RNA-seq expression profiles from 53 separate studies (15) (Fig. 1a). Each sample was processed through a standardized data processing pipeline to assess the data quality and filter out poor quality profiles (See Methods, Fig. 1b) (14). The final compendium was composed of 647 high quality expression profiles, spanning 231 unique conditions that describe M. tuberculosis’s response to various nutrient sources, stressors, antibiotics, and infection events. After the final compendium was obtained, a previously developed ICA algorithm was used to decompose the data into 80 robust iModulons (16) (Fig. 1a).

In order to provide biological interpretation of the results, iModulons were categorized by associating the set of genes in each iModulon to knowledge types, including TF binding sites, KEGG pathways, GO terms, and other associable knowledge found in the literature. Due to the variances in TF binding site data across various databases and studies, a new set of literature TRN annotations for M. tuberculosis TF regulation and binding was constructed by compiling information across 42 different databases and studies (Supplemental Data Set S2). Among the 42 sources, a majority of TF binding sites used in this study were obtained from the TB database (http://tbdb.bu.edu/tbdb_sysbio/MultiHome.html) published by Galagan et al. and the MTB Network Portal (2, 4, 7). An iModulon was considered associated with a particular knowledge type if there was a statistically significant (FDR < 0.01) overlap between the genes found in the iModulon and the knowledge type (See Methods). Some iModulons were manually annotated due to shared functions of constituent genes, or presence of deleted genes (See Methods). iModulons that share a statistically significant overlap with known regulons can further be classified based on the number of shared genes between the two clusters and the relative size of both the iModulon and the regulon (Fig. 1d). iModulons can be classified as “Well Matched,” a “Subset” of the regulon, contain mostly genes that were “Previously Unknown” to be within the regulon, or “Poorly Matched.”

10.1128/msphere.00033-22.1 DATA SET S1 Description of additional citations used to compile analyzed data set. Download Data Set S1, XLSX file, 0.01 MB. Copyright © 2022 Yoo et al. 2022 Yoo et al. https://creativecommons.org/licenses/by/4.0/ This content is distributed under the terms of the Creative Commons Attribution 4.0 International license.

10.1128/msphere.00033-22.2 DATA SET S2 Description of citations used to compile transcriptional regulatory network referenced in this study. Download Data Set S2, XLSX file, 0.01 MB. Copyright © 2022 Yoo et al. 2022 Yoo et al. https://creativecommons.org/licenses/by/4.0/ This content is distributed under the terms of the Creative Commons Attribution 4.0 International license.

ICA also captured the activity of each iModulon in each sample, which were used to examine the response of M. tuberculosis to various environments. In order to minimize batch effects between the 53 studies, activity levels for each project were centered to a reference condition within the experimental subset (17). By reconstructing the original expression data set using only the gene weights and activities of individual iModulons, we can calculate the explained variance of each iModulon and provide a measure of how important each one is in the data set (Fig. 1e). The iModulon with the highest contribution to expression variation is one of two associated with DevR, a hypoxia onset transcriptional regulator. Altogether, the 80 iModulons account for 61% of the total variance in the compendium, which is comparable to, but slightly lower than similar decompositions in other organisms (9–11), which range from 68% to 76%. One possible reason for the decrease in explained variance for this organism is the more complex protein-DNA interactions in M. tuberculosis, which include many seemingly inactive, nonregulatory binding events (7). Another is the particular condition space explored by the M. tuberculosis literature, which emphasizes infection models and redox perturbations that do not typically decompose as well as controlled monococulture conditions and perturbations to more transcriptomically simple systems.

After examining the mapped knowledge types and iModulon activities, each iModulon was assigned a functional category (Fig. 1f). Most categories indicated a specific biological function, such as ‘Redox’, ‘Virulence/Persistence’, ‘Nucleic Acid’, and ‘Antibiotic’. We also included three technical categories. For example, the ‘Unknown Function’ category contains iModulons that have been mapped to an established TF regulon, but the function of the TF remains unclear. “Uncharacterized'' iModulons are those which had little overlap with known TFs or knowledge types, but still contained a significant number of genes. Finally, “Single Gene” iModulons are those that primarily track the expression of a single gene, and are treated as an artifact of the ICA decomposition (16). It is important to note that ‘Single Gene’ iModulons are so named based on the presence of exactly one outlier gene weight, but our automated threshold assignment may include additional genes due to skewness in the gene weight distributions resulting from slight correlations in expression. Thus, ‘Single Gene’ iModulons may contain more than one gene, as long as only one gene has significantly higher weighting.

We generated searchable, interactive dashboards for each iModulon and gene in our compendium, which are available at iModulonDB.org (18). Since this genome-scale TRN covers all publicly available high quality transcriptomic data as of August 20, 2020, other researchers are encouraged to use this site to explore the genes and regulators of interest to them.

The ICA decomposition resulted in: (i) the identification of 80 sets of independently modulated sets of genes across the entire compendium (i.e., the iModulons), dramatically reducing the dimensionality of the 3,906-gene transcriptome, 2) the catalog of the iModulon activities under the 657 conditions, and 3) the functional annotations to the iModulons, resulting in a knowledge-based description of the majority of the variation in the compendium.

### iModulons capture the activity of known transcriptional regulators VirS and Zur.

**FIG 2:**
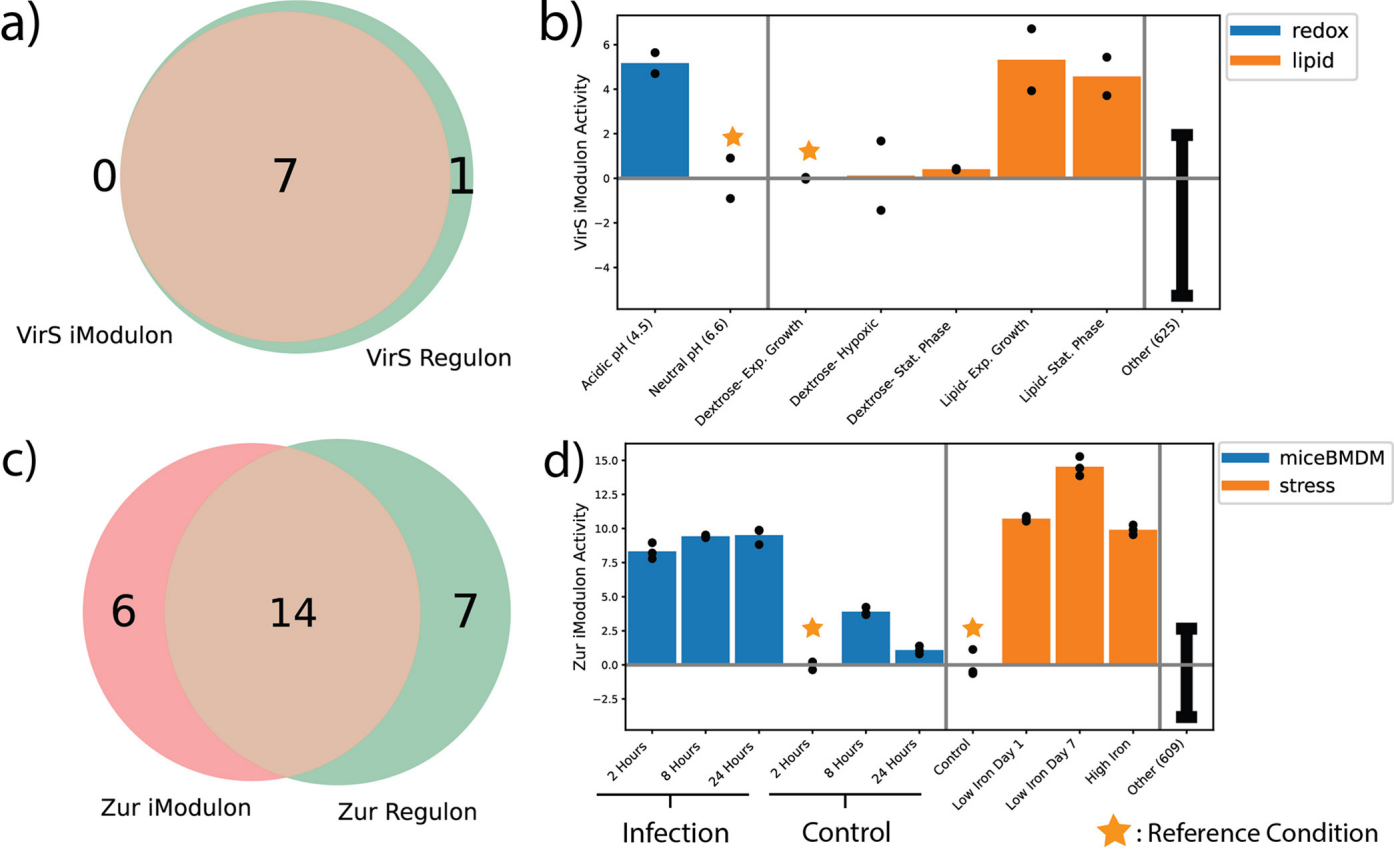
iModulons Capture Activity of Known Transcriptional Regulators Zur and Lsr2. (A) Venn diagram showing the genes that overlap between the established Zur regulon and the calculated iModulon. (B) Bar plot representing the activity of the Zur iModulon across infection, high iron, and low iron conditions. In general, iModulon activity corresponds with expression of the genes within that iModulon, with positive activity representing increased expression. (C) Venn diagram showing the genes that overlap between the established Lsr2 regulon and the calculated iModulon. (D) Bar plot representing the activity of the Lsr2 iModulon across three different infection conditions (THP-1 macrophages, mice bone marrow derived macrophages (miceBMDM), and mice neutrophils (miceNF)). *For activity bar plots*, *error bars represent mean and standard deviation of all other samples*, *black dots represent the activity of each replicate for a condition*, *and vertical gray bars separate the samples into projects. Each project is normalized to a reference condition within that project such that the reference condition represents zero activity*.

Two iModulons captured the actions of the VirS and Zur regulons, respectively (Fig. 2a and c). These iModulons provide a good example of how iModulons complement regulons by recapitulating expected regulator activity. In M. tuberculosis, the VirS TF has been identified as an AraC family transcriptional regulator that regulates the *mymA* operon, is sensitive to acidic pH environments, and plays a role in the modification of fatty acids required for the cell membrane (19). Examining our iModulons, we find one gene cluster with statistically significant overlap with the known VirS regulon, as all 7 genes found in the iModulon can also be found in both the 8 gene regulon and the *mymA* operon (Fig. 2a). This near perfect match between the iModulon and the known regulon strongly suggests that the activity of this iModulon under various conditions would correlate with prior experiments, and thus we examine the activity of the VirS iModulon. We found that the activity of the VirS iModulon was significantly upregulated under acidic conditions compared to a neutral pH control, which matches prior findings that demonstrated upregulation of the *mymA* operon under acidic pH due to *virS* regulation (19). Given the additional role of the *virS* TF in the modification of fatty acids for the cell membrane and the known accumulation of C24/C26 fatty acids in *virS* knockout strains, we also checked to see if the activity of the iModulon reflects the TF’s association with fatty acids (20). Within our data set, we found one study of M. tuberculosis in various states (exponential growth phase, stationary phase, and hypoxic) in media containing either only dextrose or only fatty acids and cholesterol. We see that VirS was upregulated in the lipid conditions compared to the dextrose control (Fig. 2b). The upregulation was greatest for exponential-phase M. tuberculosis grown in lipid only media, which makes sense if *virS* plays a role in modifying lipid membrane. Overall, the VirS iModulon captures not only the known genes of the regulon, but reflects the expected activity of the regulon.

In addition to the VirS iModulon, we found another iModulon that had significant overlap with the Zur regulon, thus leading us to label it as the Zur iModulon (Fig. 2c). Zur is a zinc-responsive transcription factor that regulates zinc homeostasis, which is significantly perturbed in the phagosome during infection events (21, 22). While the Zur iModulon does not have complete overlap with the known regulon as the VirS iModulon did, we still find that the activity of the iModulon reflects the behavior of the TF. The Zur iModulon was highly upregulated in *in vitro* macrophage infection conditions compared to controls, showing that the Zur iModulon quantitatively captured the previously reported derepression of the Zur TF under those conditions (Fig. 2c) (22). Interestingly, while Zur is typically activated by zinc ions, the Zur iModulon exhibited high activities when iron concentrations deviated greatly from standard media. This observation matches previous studies that detail how the ESX-3 secretion systems regulated by Zur play a small role in maintaining iron homeostasis in tandem with the iron uptake regulator, IdeR (23, 24).

Overall, both the VirS and Zur iModulons were able to capture the known activities of their associated TF, and many other iModulons matched the known regulators with similarly high recall (Fig. 1d). This evidence suggests that the calculated iModulons provide a quantitative structure that largely agrees with the known TRN of M. tuberculosis.

### iModulons support the predicted function of the uncharacterized transcription factor Rv0681.

Since iModulons successfully captured the structure and function of the known M. tuberculosis TRN, we further investigated if iModulons could be used to elucidate functions for TFs which have yet to be fully explained. Therefore, we examined the activity of the Rv0681 iModulon to determine the function of the associated TF.

**FIG 3:**
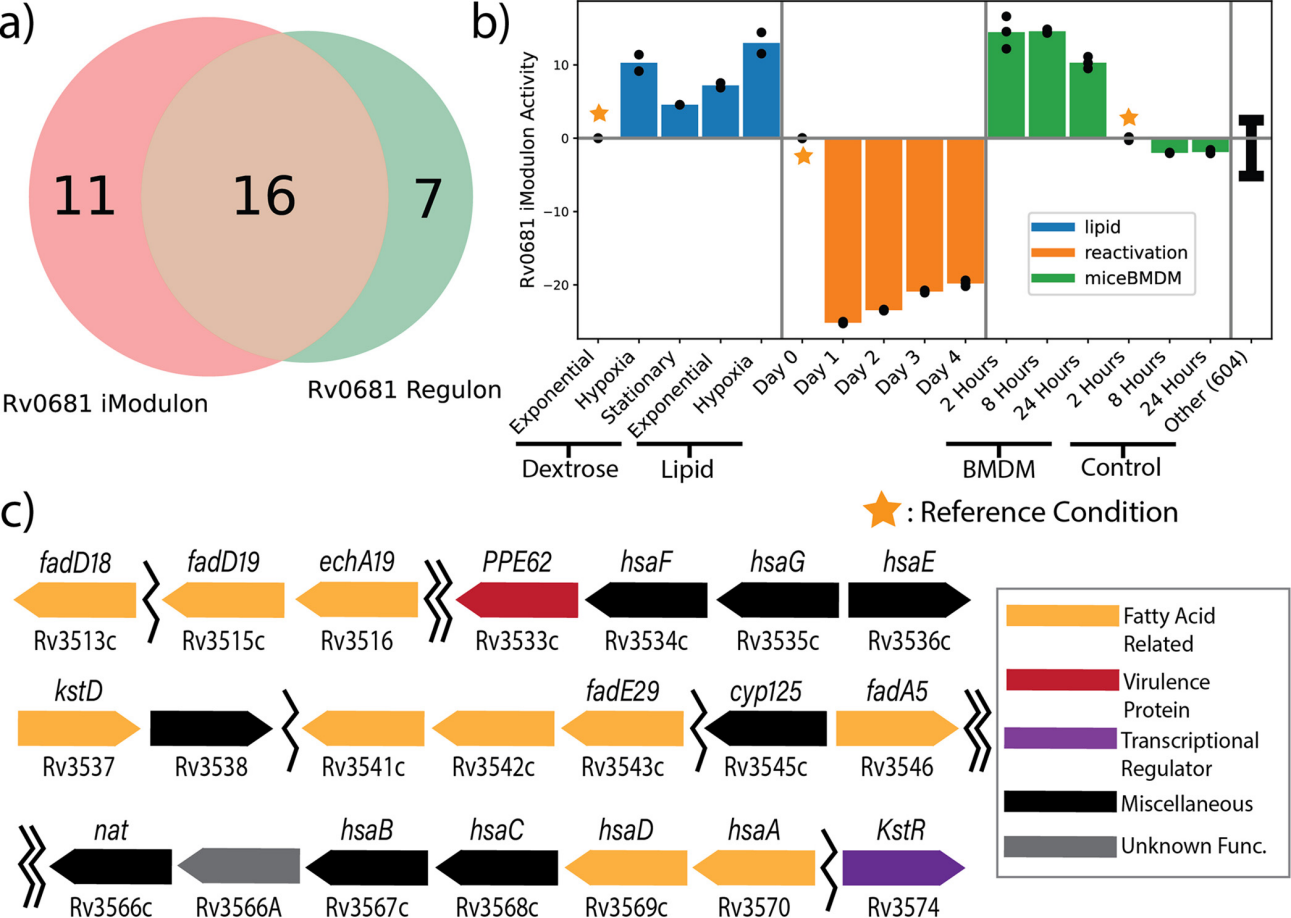
Functional Characterization of Rv0681 and involvement in lipid metabolism. (A) Venn diagram displaying the genes that overlap between the predicted Rv0681 regulon and the calculated Rv0681 iModulon. (B) Barplot displaying the activities of the Rv0681 iModulon across lipid, hypoxic reactivation, and infection conditions. (C) A diagram that characterizes the position and function of the genes found in the Rv0681 iModulons. Many of these genes are related to fatty acids and cholesterol, including the KstR transcription factor (27, 79, 80). Single jagged lines indicate a small skip between two iModulon genes (less than 10 genes), while double jagged lines indicate larger skips.

Rv0681 is a HTH-type transcriptional regulator that has been experimentally shown to be phosphorylated by the PknH kinase, though not much more is known about the function of the TF (25, 26). The Rv0681 iModulon had significant overlap with a previously described Rv0681 regulon, and thus was a candidate for functional discovery (Fig. 3a) (2, 4). While previous definitions of the Rv0681 regulon have suggested that the TF is related to lipid transport and metabolism, the inclusion of additional genes in the iModulon bearing a similar Cluster of Orthologous Genes (COG) classification supports that role for the TF (Fig. 3b) (7). Among these newly included iModulon genes was the KstR TF, an important regulator for cholesterol metabolism in M. tuberculosis (27). Given that the KstR TF regulates many genes associated with cholesterol catabolism similar to the ones found within the Rv0681 iModulon, the fact that our data suggest co-stimulation of the two TF’s suggests to us Rv0681 is an important transcriptional regulator for lipid and cholesterol metabolism (27, 28). It may even be possible that Rv0681 may help regulate the expression of the KstR TF, but further investigation is required to elucidate any possible regulatory mechanisms.

The activity of the Rv0681 iModulon also supports its role in lipid catabolism. In one project, M. tuberculosis was grown on either dextrose or lipid-only media, during exponential-phase, stationary-phase, and hypoxic exposure (BioProject: PRJNA390669) (29). We found that using lipid as a carbon source led to a significant upregulation of the iModulon relative to dextrose, regardless of growth phase (Fig. 3c). This would be consistent with a function in lipid catabolism.

In a second data set, M. tuberculosis was first induced into a persistence state via hypoxia. The bacteria was then reactivated via reaeration, and RNA-Seq was performed once a day for 4 days (BioProject: PRJNA327080) (24). The Rv0681 iModulon had significantly decreased activity when reactivating from dormancy (Fig. 3c), suggesting that Rv0681 is important for hypoxia and dormancy response, but is downregulated when ample oxygen is available.

Due to the close relationship between lipids, hypoxia, and infection, we examined a third data set that tested the infection of mouse BMDM (BioProject: PRJNA478245) (22). The iModulon was significantly upregulated during infection of the macrophage compared to noninfection controls at all time points, suggesting that the iModulon is involved with infection as well. This supports findings from the same study which suggested that lipid metabolism for cell wall remodeling was an essential component of transcriptional remodeling during infections. Altogether, we propose that Rv0681 is a transcription factor that regulates lipid metabolism (likely lipid catabolism) to promote survival in stressful conditions such as hypoxia and infection.

### Redefining the core lipid response in M. tuberculosis.

While individual iModulons can provide information about a single TF, one of their most useful functions is to simplify analysis of organism-wide transcriptional responses. Given the association between Rv0681 and lipid metabolism, we were interested in determining which other iModulons were activated under lipid-rich conditions. Within the compendium, a study examined the differentially expressed genes between dextrose and lipid-fed M. tuberculosis across 3 metabolic states (exponential growth, stationary phase, hypoxia) (BioProject: PRJNA390669) (29). The study then defined a “core lipid response,” which contained genes that were found to be differentially expressed between dextrose and lipid media across all three metabolic states. This core lipid response was composed of 6 genes: Rv3161c, Rv3160c, Rv0678, Rv1217c, PPE53 and *che1* (29). Since a core lipid response can be crucial for identifying potential targets to combat M. tuberculosis infections, we were interested if iModulons could be used to define a regulator-level core lipid response utilizing the same RNA-seq data.

**FIG 4:**
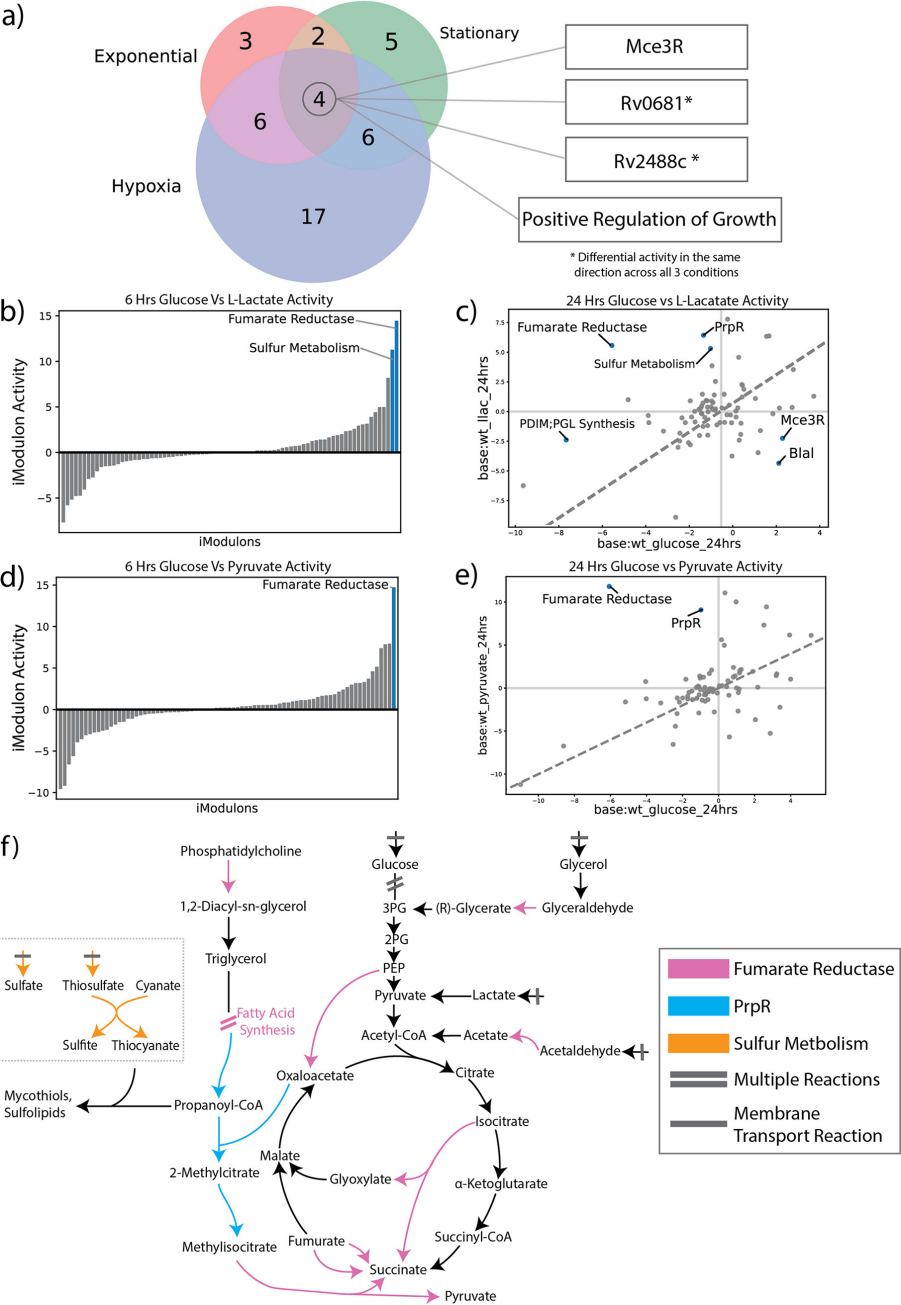
iModulons Illuminate Metabolic Shifts from Changes in Carbon Source. (A) A three-way venn displaying the differentially activated iModulons between dextrose and lipid conditions across three metabolic states (exponential, stationary, and hypoxia). The iModulons that were differentially activated across all three states represent the core lipid response. (B) A 1D DIMA plot representing the differentially activated iModulons at 6 h between L-lactate and glucose conditions. (C) DIMA plot representing the differentially activated iModulons at 24 h between L-lactate and glucose conditions. (D) A 1D DIMA plot representing the differentially activated iModulons at 6 h between pyruvate and glucose conditions. (E) DIMA plot representing the differentially activated iModulons at 24 h between pyruvate and glucose conditions. (F) A metabolic map representing the reactions controlled by differentially activated iModulons across carbon source shifts. Arrows represent reactions between metabolites, and reactions with bars represent transport from the environment. Map displays how reactions controlled by the significant iModulons are connected to one another, and in conjunction with DIMA plots can describe potential changes in metabolite flux. For example, the Fumarate Reductase iModulon is differentially upregulated across all time points and carbon sources, which would tend to increase the amount of enzyme present and ultimately catalyze higher flux through the pink pathways (in the absence of protein and metabolite-level regulation, which cannot be studied with our data).

iModulon activities were examined between lipid and dextrose conditions, and iModulons with significant differential activity (iModulon activity change > 5 and FDR < 0.01) across all three metabolic states were labeled as part of the new core lipid response (Fig. 4a). While the original study identified a core lipid response composed of only 6 genes, our analysis of the same data identified a core lipid response of four iModulons: Mce3R, Rv0681, Rv2488c, and Positive Regulation of Growth (PROG) (Supplemental Data Set S3). Altogether, these four iModulons contained 80 genes. As stated before, the Rv0681 contains many genes associated with lipid and cholesterol catabolism, and the Mce3R TF is known to regulate operons associated with beta-oxidation, propanoate metabolism, and other lipid related processes (30). On the other hand, PROG was labeled based on its significant overlap with the KEGG pathway of the same name, and contains genes associated with transcriptional regulation, translation, and cell cycle control, particularly during *in vitro* growth (31). Rv2488c contains a variety of genes of different functions, and it is role in the core lipid response will be explained in greater detail later in this paper. The genes contained in Rv0681 and Mce3R makes it clear that they would be involved in the catabolism and metabolism of the lipid carbon sources, while the PROG iModulon may be triggered by the lipid rich environments to transition the organism into a reduced growth state, possibly similar to those found during *in vitro* growth (evidenced by similar differential iModulon activity in other infection conditions). Within the lipid study, the Rv0681 and Rv2488c iModulons had consistent activation across all three cell states, whereas Mce3R and PROG were found to have both increased and decreased activity depending on the cell state. Though the activities of Mce3R and PROG vary, we maintain that all four of these iModulons are important systems for M. tuberculosis in a lipid rich environment.

10.1128/msphere.00033-22.3 DATA SET S3 iModulons that compose the core lipid response. Download Data Set S3, XLSX file, 0.01 MB. Copyright © 2022 Yoo et al. 2022 Yoo et al. https://creativecommons.org/licenses/by/4.0/ This content is distributed under the terms of the Creative Commons Attribution 4.0 International license.

Upon closer examination, we found that five of the six genes previously identified as part of the core lipid response were captured by the Rv2488c iModulon, whereas *che1* was not found in any of the computed iModulons. Besides the five core lipid genes, the Rv2488c iModulon also contains various transcriptional regulators and membrane-associated proteins, such as the MmpS4, MmpL5, and MmpS5 efflux pumps. It is important to note that this iModulon was named after Rv2488c because it captured all three genes that Rv2488c was known to regulate, but its other 9 genes are not known to be regulated by it. The coregulation of these important functions may be due to costimulation across all available RNA-seq profiles, or point to an important knowledge gap about the regulation of the lipid response. Further studies should examine the possible role of Rv2488c as a regulator for the transport of lipids in and out of the cell, with an additional potential role in modulating essential, lipid-activated cellular defense (32). Taken together, the results show that iModulons provide a modular definition of a core lipid response, propose uncharacterized regulators of interest to that response, and add to our knowledge on how M. tuberculosis responds to lipids.

### iModulons elucidate transcriptional responses to shifts in carbon sources.

Given the transcriptomic response M. tuberculosis exhibited when grown with lipids as a sole carbon source, we were interested to see how the organism would respond to other carbon sources. In order to study such effects, we utilized data obtained from a study where either glucose, lactate, or pyruvate was used as a sole carbon source (BioProject: PRJNA480455) (33). In total, the study contained six different conditions, representing the three carbon sources (glucose, lactate, and pyruvate) with two time points each (6 h and 24 h). The original study found that genes associated with the glyoxylate shunt and Krebs cycle, such as *pckA* and *icl1*, were essential and highly expressed in lactate and pyruvate conditions. To assess if iModulons could capture the upregulation of the genes highlighted in the previous findings, we created several DIMA (Differential iModulon Activity) plots to examine which iModulons had significantly different activities between glucose and the alternate carbon source (Fig. 4b to e). Three iModulons were of particular interest: Fumarate Reductase, Sulfur Metabolism, and PrpR (Supplemental Data Set S4).

10.1128/msphere.00033-22.4 DATA SET S4 iModulons that were found to be differentially regulated across changes in carbon sources. Download Data Set S4, XLSX file, 0.01 MB. Copyright © 2022 Yoo et al. 2022 Yoo et al. https://creativecommons.org/licenses/by/4.0/ This content is distributed under the terms of the Creative Commons Attribution 4.0 International license.

For cells growing on both lactate and pyruvate, the Fumarate Reductase iModulon was upregulated at all time points compared to the glucose-fed conditions. The Fumarate Reductase iModulon contained 33 genes associated with the TCA cycle and fatty acid synthesis, including *icl2*, *pckA*, and *fad* genes (Fig. 4b). Many of the genes in this iModulon were also highlighted by the original study for survival in lactate and pyruvate media, which include genes that regulate the glyoxylate shunt. However, the Fumarate Reductase iModulon also captures the expression dynamics of many genes not found in the original research. These include the *fad* genes, which code for various enzymes in fatty acid synthesis, the *yrbE1* putative permeases, and the *mce1R* transcription factor, which has an important role in establishing the persistence state *in vivo* (34, 35). Many of these genes are important for maintaining lipid homeostasis, which suggests that the systems that help metabolize pyruvate and lactate are transcriptionally connected to the same systems that metabolize or synthesize lipids (36). Additionally, these metabolites may play a role in the organization of granulomas and the persistence state, based on the coregulation with Mce1R.

When lactate is used as a carbon source, we observed very strong upregulation of the Sulfur Metabolism iModulon. This is interesting given that sulfur homeostasis should not have been perturbed by the change to the media. Sulfur is essential for the production of mycothiol, which maintain redox homeostasis in *Actinobacteria* (37, 38), an essential function for survival in a host. Indeed, the only condition that creates a stronger activation for this iModulon is a redox stress condition (39). We therefore propose an important link between lactate and sulfur metabolism. It may be explained by changes to sulfurous amino acid metabolism, reactive oxygen species accumulation under lactate oxidation, or a more distal causation: given that lactate is a major carbon source during infection (40), it may be a cue for the host cell environment which M. tuberculosis treats as a signal to prepare for redox stress.

We also found evidence of time-dependent iModulon responses during exposure to alternative carbon sources. At 24 h, we found significant upregulation of the PrpR iModulon under both lactate and pyruvate conditions (Fig. 4b and d). In M. tuberculosis, the PrpR TF is responsible for control of the *prp* operon, which codes for several key enzymes that break down Propionyl-CoA into pyruvate and succinate, which can be used in the methylcitrate cycle to produce NADH (Fig. 4f) (41). The appearance of the PrpR iModulon at 24 h and not at 6 h suggests that this is a starvation response, and we hypothesize that the iModulon is activated to supplement the production of NADH and ATP from solely lactate carbon sources.

Overall, the use of iModulons and their associated activities to elucidate systematic changes in M. tuberculosis under different carbon sources is effective. Here, we were able to highlight insights into which portions of carbon metabolism were coregulated (such as the surprising relationship between the TCA cycle and fatty acid synthesis), as well as when they are used (the activation of the PrpR iModulon as a possible starvation response).

### iModulon analysis of time-course data agrees with prior models of TF responses to hypoxia.

**FIG 5:**
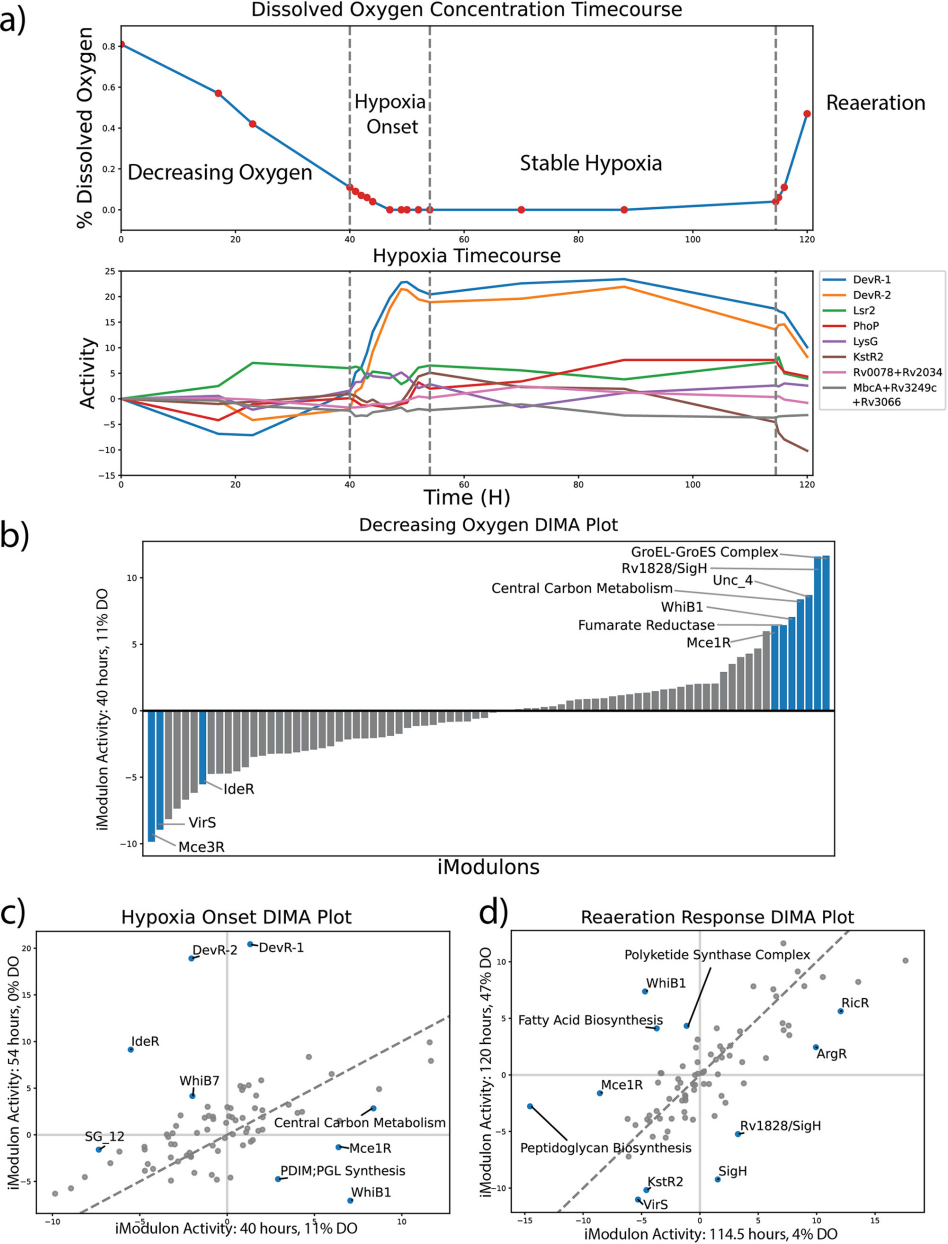
iModulons help Categorize the Phases of Hypoxia Response, including Metabolic Anticipation. (A) Time Course of M. tuberculosis undergoing Decreasing Oxygen, Hypoxia Onset, and Reaeration. The top plot displays the dissolved oxygen concentration in the environment, and the bottom plot displays the activities over time for iModulons controlled by TFs previously identified to be highly involved in hypoxic response (2). The TF Rv2034 is represented by the iModulon Rv0078+Rv2034 and Rv3249c is represented by MbcA+Rv3249c+Rv3066 iModulons. (B) DIMA plots of hypoxia phases were created by comparing the iModulon activities between the first and last time point of each phase. The bar graph represents a 1D DIMA plot for the decreasing oxygen phase, since the original *t* = 0 time point served as the reference condition. (C) DIMA plot for the Hypoxia Onset Phase. (D) DIMA plot for the Reaeration phase.

We analyzed the important iModulons and significant activities during a hypoxia time course study in our compendium by Peterson, et al. (BioProject: PRJNA478238) (22). During this study, the organism was exposed to changing dissolved oxygen levels, and we categorized the changes into four temporal phases: (i) Decreasing Oxygen, 2) Hypoxia Onset, 3) Stable Hypoxia, and 4) Reaeration. (Fig. 5a). The transcriptional changes associated with hypoxia are relatively well-characterized in M. tuberculosis, and thus we assessed if the activities of the iModulons would recapitulate previous studies (Supplemental Data Set S5) (2). The prior study proposed a model of the M. tuberculosis TRN and determined that the DevR (also called DosR) and Rv0081 TFs serve as the primary regulators for the hypoxic response, while other TFs such as Rv2034, Rv3249c, KstR, and PhoP can alter the response. In order to test that the iModulons recapitulate the prior model, we examined iModulons mapped to hypoxia-associated transcriptional factors and examined their activities throughout the hypoxic time course study. We found that the DevR, PhoP, KstR2, and Lsr2 iModulons had increased activity during the hypoxia time course (Fig. 5b). The two DevR iModulons showed the highest activity during the Hypoxia Onset phase, which recapitulates the previous understanding that the DevR TF controls the hypoxia onset response (Fig. 5a) (42). A primary component of both DevR iModulons are the nitrate reductase and nitroreductase genes, which are associated with the reduction of the electron transporters NADH and NADPH. The importance of these electron transporters give further evidence that the DevR iModulons recapitulate the hypoxia onset response in M. tuberculosis.

Additionally, the increase in activity of the Lsr2, KstR2, and PhoP iModulons also capture the known transcriptional changes associated with hypoxia. Due to the lack of a KstR iModulon, KstR2 activity was examined instead as both iModulons are thought to regulate cholesterol metabolism, which may be important for a hypoxia response (2). The Rv0078+Rv2034 and MbcA+Rv3249c+Rv3066 iModulons were not significantly expressed at any point in the time course.

### Different levels of oxygen lead to distinct transcriptional states.

After confirming that our iModulons are consistent with our current understanding of hypoxia, we examined the activities of the iModulons in a phase-specific manner across three of the four phases. DIMA plots were created to compare the iModulon activities from the first and last time point of each phase, and the significant iModulons were examined (Fig. 5c and d). We chose not to analyze the iModulons during stable hypoxia given that there were limited significant changes in iModulons. The lack of change in the transcriptome is consistent with the dormant persistence state that hypoxia induces (43).

Here, we define the Decreasing Oxygen phase to represent the time when dissolved oxygen levels transition from 81% to 11%. Examination of significant iModulons during this phase reveals a three part response (Fig. 5b). The first response is the significant increase in the production of enzymes associated with central carbon metabolism and energy production, and is captured by the Central Carbon Metabolism and Fumarate Reductase iModulons. The second response was an increased activity in growth and cell replication systems, which was captured by the upregulation of the Rv1828/SigH, GroEL-ES complex, and WhiB1 iModulons. Rv1828/SigH contains genes that encode a wide range of proteins, including cell division proteins (SepF, FtsZ), DNA helicases (RuvA/B/C), and DNA polymerases (44). Additionally, we found both the WhiB1 and GroEL/ES complex iModulons play a role in protein synthesis. WhiB1 also contains several genes that code for RNA polymerase subunits, and is likely a translation iModulon that has been seen in the ICA decompositions of other organisms (9, 10, 45). All three iModulons are related to growth and replication, which suggests that cell division is an important response in M. tuberculosis in a decreasing oxygen environment. Though surprising given the relatively decreased metabolic efficiency in low oxygen environments, this may be explained by a decrease in oxidative stress or an evolutionary advantage for strains that replicate as much as possible prior to entering dormancy.

The final response of the Decreasing Oxygen phase was a shift in the mammalian cell entry (Mce) proteins produced within the cell. This response is captured by increased activity in the Mce1R iModulon and a decrease in activity for the Mce3R iModulon. The Mce proteins are cell surface proteins that are thought to play a role in lipid transport, redox reactions, and invasion of host cells (30, 34, 46, 47). Further examination of the Mce1R and Mce3R iModulons indicates that as the time course proceeds and the cell enters Hypoxia Onset and Stable Hypoxia, the activities of the two iModulons returned to their original reference state; the activity of the Mce3R iModulon significantly increases while the activity of the Mce1R iModulon significantly decreases. Given the close relationship between hypoxia, infection events, and activity levels over this experiment, we predict that proteins in the Mce1 iModulon help facilitate the initial stages of infections while proteins in the Mce3 iModulon facilitate cell entry into a dormant state.

The next phase of the hypoxia time course was the Hypoxia Onset phase, where the dissolved oxygen levels decrease from 11% to 0% (Fig. 5c). Apart from the previously described activities of both DevR iModoulons, we also found that a few of the iModulons had inverted activities during Hypoxia Onset compared to the Decreasing Oxygen phase. The Mce1R, WhiB1, and Central Carbon Metabolism iModulons showed decreased activity over the course of the Hypoxia Onset phase. These decreases are consistent with a more dormant, less metabolically active persistence state. On the other hand, the IdeR iModulon moved from a decrease in activity in the prior phase to a significant increase in activity during Hypoxia Onset. Additionally, we found two iModulons, the WhiB7 and PDIM;PGL Synthesis iModulons, with significant changes in activity during this phase. WhiB7 is a redox homeostasis transcriptional regulator that has also played a role in drug resistance (48). The PDIM;PGL Synthesis iModulon captures genes associated with the production of phthiocerol dimycocerosate (PDIM) and phenolic glycolipids (PGL). These families of molecules have been associated with cell wall impermeability, phagocytosis, defense against nitrosative and oxidative stress and possibly, biofilm formation (49). The presence of both these systems during hypoxia is expected, though we did not expect PDIM;PGL Synthesis to have decreased activity during Hypoxia Onset. This would suggest that while PDIM and PGL molecules are important for oxidative stress defense, their production may require more energy than can be generated in an anaerobic environment or are otherwise detrimental to the survival of the cell.

The final phase of the hypoxia time course was the Reaeration phase (Fig. 5d). During this phase, the cell returns to an aerobic environment as dissolved oxygen levels increase from 0% to 47%, and we found significant changes in several iModulons. Most interesting among these are the Peptidoglycan Biosynthesis and Polyketide Synthase Complex. In M. tuberculosis, both polyketides and peptidoglycans are cell membrane bound molecules that play a role in virulence and persistence. Peptidoglycans are involved in cell growth and host response manipulation, while polyketides are essential in the formation of biofilms and are likely to improve persistence (50, 51). The increased activation of these iModulons under Reaeration suggests that M. tuberculosis attempts to defend itself from a possible host response during this phase. We also found that the Fatty Acid Biosynthesis iModulon had increased activity while KstR2 had decreased activity. Thus, we can conclude that under reaeration conditions, M. tuberculosis moves from the consumption of lipids and cholesterol to production.

Altogether, we showed that iModulons can validate previous results obtained from the hypoxia time course, while also revealing a concise summary of the complex transcriptional responses that M. tuberculosis undergoes throughout large shifts in oxygen concentration.

### M. tuberculosis has host cell-specific transcriptional responses.

**FIG 6:**
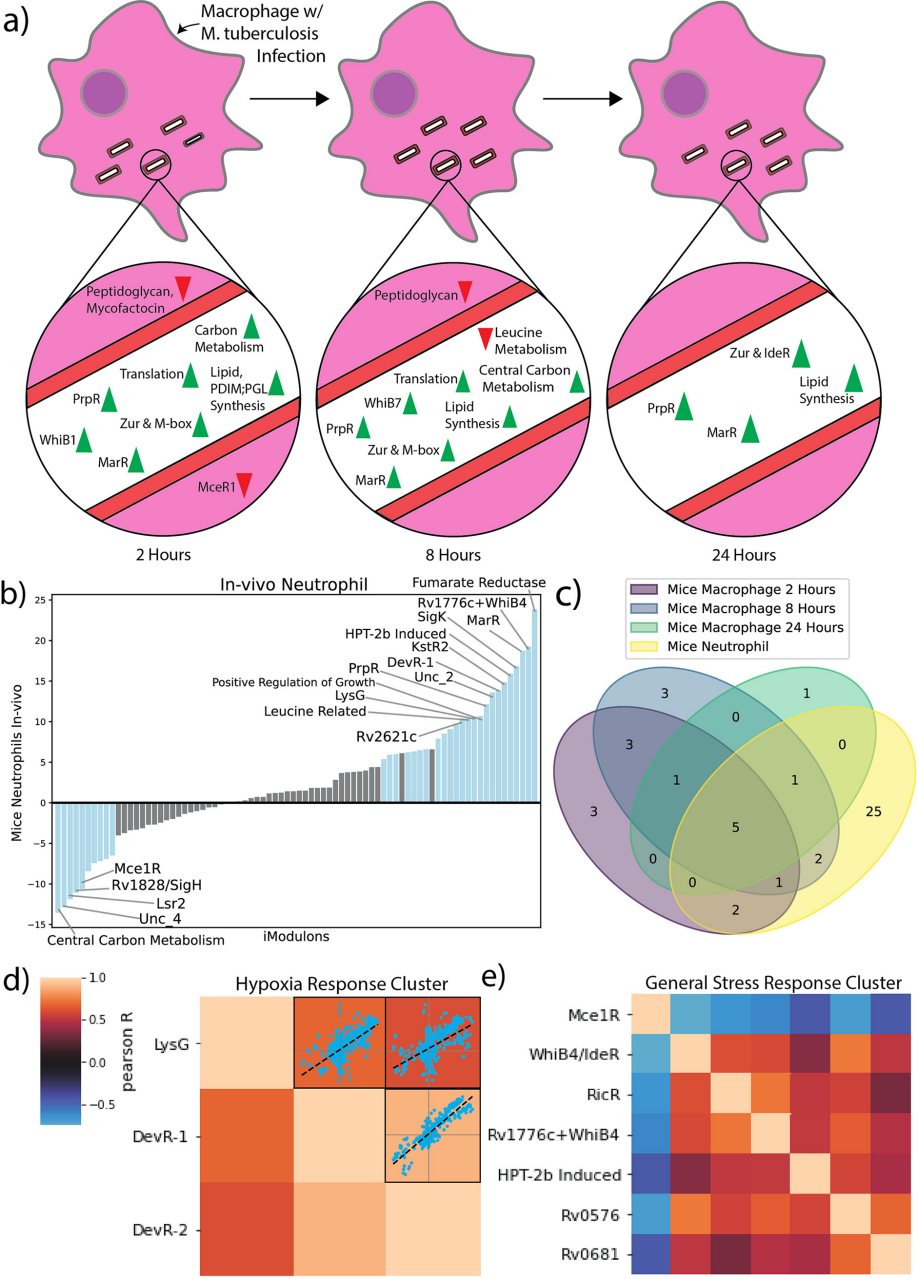
iModulon Response to Infection of Mice Macrophages and Neutrophils and Pearson R iModulon Clusters. (A) A time course of the iModulon activities during infection of mice BMDM. The iModulons with differential activities at each time point are displayed as upregulated (green) or downregulated (red). Peptidoglycan, Mycofactocin, and MceR1 are displayed outside the cell to indicate regulation of secretory pathways. (B) 1D DIMA plot of differential iModulons between control noninfectious condition and *in vivo* infection condition. Surprisingly, the most upregulated and most downregulated iModulons both regulate different portions of central carbon metabolism, which suggests that central carbon metabolism plays a large role in infection. (D) A core infection response was constructed by examining the iModulons with differential activity across all infection conditions (3 time points in mice macrophage infection and 1 neutrophil condition). The core infection response was found to consist of KstR2, MarR, PrpR, Rv0681, Uncharacterized 2, and Zur. (D) Hypoxia Response iModulon cluster calculated using Pearson R score and agglomerative clustering. Scatterplots that provide pairwise comparison of the activities of the iModulons across all experimental conditions is provided to indicate the relatively high correlation between these three iMoudlons. Color bar indicates pairwise Pearson R score. (E) General Stress Response iModulon cluster calculated from Pearson R score and agglomerative clustering.

Due to the broad pathological impact of M. tuberculosis, we additionally used iModulons to examine the transcriptional response of M. tuberculosis during infection of two different host cell types: macrophages and neutrophils. It is important to note here that while both cell types are important players in the immune system for fighting against M. tuberculosis, each cell type responds very differently when encountering the bacterium. Neutrophils serve as key mediators in the innate immune response, with 3 potential responses when encountering the bacterium: direct killing of bacteria via enzymes or reactive oxygen species, trapping of bacteria via neutrophil extracellular trap formations, and secretion of cytokines to signal other immune cells (52). On the other hand, the macrophages primarily respond to M. tuberculosis bacteria through phagocytosis. While macrophages seek to eliminate the bacteria via production of pro-inflammatory cytokines and reactive oxygen species, macrophages also serve as the primary host cell type for the bacteria (53, 54). The transcriptional response of the bacteria to each immune cell type will likely have distinct differences, and in order to investigate these potential differences we examined the activities of iModulons in two different infection data sets. In one data set, M. tuberculosis was grown *in vitro* during infection of mice bone marrow-derived macrophages (BMDM), and RNA-Seq was performed at 2, 8, and 24 h after infection (BioProject: PRJNA478245) (22). In the other data set, M. tuberculosis was grown *in vivo* with mice neutrophils, and RNA-Seq was performed at between 2 and 8 h after infection (BioProject: PRJNA588440) (55). DIMA plots were created comparing each infection condition to a control at the same time point (Fig. 6a).

Examination of the significant iModulons under the three time points of the mice BMDM conditions resulted in consistent patterns (Supplemental Data Set 6). For example, the activity of the acid-sensing MarR iModulon increased across all time points. MarR is an acid transcriptional repressor that controls the expression of virulence associated methyltransferase, and it is activation during infection events allows M. tuberculosis to adapt and replicate in acidic intracellular environments (56). In addition, we found that PrpR, lipid metabolism iModulons, along with the metal sensing Zur, M-box, and IdeR iModulons, were consistently upregulated throughout the infection time course. All of these iModulons play a role in either starvation or hypoxia response, indicating that residence within a macrophage requires distinct adaptations to multiple stresses (57, 58). Additionally, the consistent upregulation of Zur is likely due to the increased concentration of zinc ions within the phagosome during infection events, a known immune response to M. tuberculosis infection (59). iModulons that are differentially regulated at only specific time points can provide context for how M. tuberculosis behaves during macrophage infections. For example, we see that the Fumarate Reductase and Central Carbon Metabolism iModulons are upregulated during the 2 h and 8 h time points, respectively. Possible reasons for this include the organism’s need to metabolize fatty acids found within the macrophage environment, thus requiring the activation of the Fumarate Reductase iModulon, or simply an increased need for energy in order to power the infection related cellular systems.

A similar analysis of M. tuberculosis under *in vivo* neutrophil conditions revealed an altered TRN response compared to *in vitro* mice BMDM infections (Fig. 6b). Comparison of differentially activated iModulons revealed 25 additional iModulons with significant activities during infection of mice neutrophils, but not during infection of mice BMDM. These neutrophil-specific iModulons include some important regulators such as DevR-2, PhoP, Mce3R, and PROG. Interestingly, DevR-2, PhoP, and Mce3R are iModulons that were found to be important during the hypoxia time course, and all three of these TF’s play an important role in M. tuberculosis hypoxic response (30, 42, 60). Given that these 3 iModulons are uniquely significant to only the infection of mice neutrophils, this suggests that the infection of mice neutrophils exposes M. tuberculosis to greater oxidative stresses compared to mice macrophages. Additionally, the presence of the PROG iModulon during the infection of mice neutrophils and not mice macrophages suggests that the patterns of replication and growth for M. tuberculosis are different between the two cell types.

While the cell type specific iModulons can provide insights into how the organism adapts during infection events, we also discovered five iModulons that exhibited consistently significant activities across all experiments (KstR2, MarR, PrpR, Rv0681, Uncharacterized 2) (Fig. 6c). All of these iModulons, with the exception of the Uncharacterized 2 iModulon, were activated in the same direction (positive activity) across the BMDM and neutrophil conditions. Overall, these results show how M. tuberculosis has different transcriptional responses depending on the host cell type, but a core infection response is required for all infection events.

### Clustering of iModulon activities across all conditions reveal coordinated stress responses.

By investigating the iModulons across various conditions, we noticed that certain sets of iModulons activated together. To investigate which iModulons had similar activities to one another, we clustered the iModulon activities, resulting in several clusters with biologically relevant implications. One such cluster contains the DevR-1, DevR-2, and LysG iModulons (Fig. 6d) (14). Given the function of DevR and the presence of the gene Rv0081 and several oxidoreductases and formate respiration enzymes in LysG, it is clear that these iModulons comprise the main hypoxic response in M. tuberculosis (2).

Clusters also described global responses in the M. tuberculosis TRN, as shown by the General Stress Response Cluster (Fig. 6e). This cluster contained infection related iModulons such as Mce1R, metal related iModulons like RicR, and lipid metabolism iModulons such as Rv0681. We found that while six of the iModulons within the cluster were positively correlated with each other, Mce1R was found to be negatively correlated with the others, indicating that stress conditions actually downregulate predicted cell entry machinery. To help visualize which systems were controlled by this cluster, we mapped the genes within each associated iModulon to known pathways using annotations from a metabolic reconstruction (61). The reactions encoded by the iModulons in the cluster linked cholesterol-catabolism pathways to propionyl-CoA biosynthesis. Propionyl-CoA is an important precursor to both mycothiols and sulfolipids, and we found that the General Stress Response Cluster also controls pathways associated with sulfur import. The cluster also controls the production of mce1 proteins, the type 1 NADH-dehydrogenase, and metal sensing systems. Type 1 NADH-dehydrogenase is known to produce ROS species and increase oxidative stress, while metal sensing systems such as those encoded by RicR are important for protection against oxidative stress (62, 63). Given the function of these genes, we propose that this cluster represents a general stress response in M. tuberculosis, most likely related to intrahost survival. It also provides insight into the major metabolic pathways associated with stress in the organism. Though the General Stress Response Cluster represents a commonly cotranscribed set of iModulons, each one is still independently modulated; there are instances where one part of the cluster is not needed and its iModulon’s activity diverges from the rest. This example demonstrates that iModulon clustering can create a complex, hierarchical understanding of the TRN.

## DISCUSSION

Here, we utilized ICA to decompose 657 distinct RNA-Seq profiles of M. tuberculosis into 80 independently modulated sets of genes, termed iModulons. Many of these iModulons correspond to important transcription factors in the organism. Using these iModulons, we revealed putative new gene associations for previously uncharacterized regulators, 2) described the transcriptional shifts that occurred during environmental changes such as carbon source shifts, hypoxia, and infections, and 3) demonstrated the presence of large clusters of transcriptional regulons that link several important metabolic systems, including lipid, cholesterol, and sulfur metabolism.

Although all data sets analyzed in the manuscript were previously reported in separate publications, we have illustrated that combining the data together elucidates hidden common signals (i.e., iModulons) across all data sets. iModulons were used to both validate previous findings, such as the identification of DevR as a major regulator of hypoxic response, and gain more detailed insights in these data sets, such as characterizing the core lipid response at the TRN level. Many of the results derived from iModulon analysis could not be detected with traditional DEG analysis, since they required the synergistic analysis of multiple data sets to detect co-expression trends. For instance, detection of the host cell-specific transcriptional responses required the analysis of two independent data sets with different host cells using a method robust to batch effects.

We also demonstrated that iModulons are effective at providing detailed, mechanistic insights into complex transcriptional changes in M. tuberculosis’s TRN. Lipid metabolism, hypoxia protection, and host cell responses are all vital factors in the success of M. tuberculosis as a pathogen, and iModulons provided a clear model of the transcriptome changes occurring under these conditions. Additionally, compendium-wide clustering of iModulon activities outlined a set of stimulons (64), or groups of genes that respond to the same stimulus, for hypoxia and general stress response. Such stimulons, especially those that respond to infection conditions, can also provide better understanding on ways to combat the pathogen.

All results presented in the manuscript are reproducible at https://github.com/Reosu/modulome_mtb. In addition, we have provided an interactive Jupyter notebook so researchers can infer iModulon activities for any new transcriptomic data sets at https://github.com/Reosu/modulome_mtb/tree/master/analyze_new_data. Researchers can also investigate the current iModulon structure of M. tuberculosis, the iModulon activities, and the original gene expression compendium at https://imodulondb.org/dataset.html?organism=m_tuberculosis&dataset=modulome. The data presented here still has potential to reveal new insights into the function of uncharacterized transcription factors and the TRN behavior of M. tuberculosis under different conditions, and this analysis can be scaled in the future to incorporate the growth of new public data sets.

### Data availability.

The iModulons composition, activities, and the code used to generate figures and results are available on Github (https://github.com/Reosu/modulome_mtb). Detailed, curated dashboards for each iModulon and gene can be searched or browsed on iModulonDB.org under the “M. tuberculosis Modulome” data set (https://imodulondb.org/). Additional information, such as the sources used to compile the RNA-Seq and TRN data sets, can be found in the supplementary files.

## MATERIALS AND METHODS

The functions used in this study and description of the methods for compiling and processing RNA-Seq data, running ICA, and computing iModulon enrichments were adapted from Sastry et al. (14).

### Compiling all public transcriptomics data.

Using the script from Sastry et al., (https://github.com/avsastry/modulome-workflow/tree/main/1_download_metadata), we found all RNA-seq data for M. tuberculosis on NCBI SRA as of August 20, 2020. We manually selected samples that used the strain M. tuberculosis H37Rv (14).

### Processing prokaryotic RNA-seq data.

To process the complete M. tuberculosis RNA-seq compendium, we used Amazon Web Services (AWS) Batch to run a Nextflow pipeline (14, 65).

The first step in the pipeline was to download the raw FASTQ files from NCBI using fasterq-dump (https://github.com/ncbi/sra-tools/wiki/HowTo:-fasterq-dump). Next, read trimming was performed using Trim Galore (https://www.bioinformatics.babraham.ac.uk/projects/trim_galore/) with the default options, followed by FastQC (http://www.bioinformatics.babraham.ac.uk/projects/fastqc/) on the trimmed reads. Next, reads were aligned to the genome using Bowtie (66). The read direction was inferred using RSEQC (67) before generating read counts using featureCounts (68). Finally, all quality control metrics were compiled using MultiQC (69) and the final expression compendium was reported in units of log-transformed Transcripts per Million (log-TPM).

### Quality control and data normalization.

To guarantee a high quality expression compendium for M. tuberculosis, data that failed any of the following four FASTQC metrics were discarded: per base sequence quality, per sequence quality scores, per base n content, and adapter content. Samples that contained under 500,000 reads mapped to coding sequences were also discarded. Hierarchical clustering was used to identify samples that did not conform to a typical expression profile.

Manual metadata curation was performed on the data that passed the first four quality control steps. Information about the strain description, base media, carbon source, treatments, and temperature were pulled from the literature. Each project was assigned a short unique name, and each condition within a project was also assigned a unique name to identify biological and technical replicates. After curation, samples were discarded if (a) metadata was not available, (b) samples did not have replicates, or (c) the Pearson R correlation between replicates was below 0.95. Finally, the log-TPM data within each project was centered to a project-specific reference condition.

### Computing the optimal number of robust independent components.

To compute the optimal independent components, an extension of ICA was performed on the RNA-seq compendium as described in McConn et al. (16).

Briefly, the scikit-learn (v0.23.2) (70) implementation of FastICA (13) was executed 100 times with random seeds and a convergence tolerance of 10^−7^. The resulting independent components (ICs) were clustered using DBSCAN (71) to identify robust ICs, using an epsilon of 0.1 and minimum cluster seed size of 50. To account for identical with opposite signs, the following distance metric was used for computing the distance matrix:

$$d_{x,y}=1-||\rho_{x,y}||$$

where *ρ_x_*_,_*_y_* is the Pearson correlation between components *x* and *y*. The final robust ICs were defined as the centroids of the cluster.

Since the number of dimensions selected in ICA can alter the results, we applied the above procedure to the M. tuberculosis compendium multiple times, ranging the number of dimensions from 10 to 320 with a step size of 20. To identify the optimal dimensionality, we compared the number of ICs with single genes to the number of ICs that were correlated (Pearson *R* > 0.7) with the ICs in the largest dimension (i.e., final components). We selected the number of dimensions where the number of non-single gene ICs was equal to the number of final components in that dimension.

### Compiling gene annotations.

The gene annotation pipeline can be found at https://github.com/SBRG/pymodulon/blob/master/docs/tutorials/creating_the_gene_table.ipynb. Gene annotations were pulled from AL123456.3. Additionally, KEGG (72) and Cluster of Orthologous Groups (COG) information were obtained using EggNOG mapper (73). Uniprot IDs were obtained using the Uniprot ID mapper (74), and operon information was obtained from Biocyc (75). Gene ontology (GO) annotations were obtained from AmiGO2 (76). The known transcriptional regulatory network was obtained primarily from the Galagan TB database and MTB Network portal databases (2, 4).

### Computing iModulon enrichments.

iModulon enrichments against known regulons were computed using Fisher’s Exact Test, with the false discovery rate (FDR) controlled at 10^−5^ using the Benjamini-Hochberg correction. Fisher’s Exact Test was used to identify GO and KEGG annotations as well, with an FDR < 0.01.

### Calculating differentially expressed iModulons across conditions.

The difference in activity of iModulons were compared across relevant conditions and significantly changed iModulons were calculated utilizing a log-normal probability distribution. For each comparison, we computed the absolute difference in the mean iModulon activity and compared it to an iModulon's log-normal distribution (calculated between biological replicates). *P* value statistics was obtained for a given pair of conditions across all iModulons and a FDR was calculated. iModulon changes were considered significant if the difference was greater than 5 and FDR < 0.01.

DIMA scatterplots compare the activities of iModulons under one condition versus another, and allow for the visualization of significantly changed iModulons. 1D DIMA plots plot iModulons under one condition to a reference condition. Reference conditions have been normalized to have 0 activity across all iModulons, and thus a bar plot is used instead of a scatterplot.

### Calculating iModulon activity clusters.

The activities of iModulons were clustered using a Seaborn clustermap (77). Pearson R correlation was used as a distance metric, and pairwise distances for each iModulon were calculated. After creation of the clustermap, the scikit-learn agglomerative clustering function was performed on the clustermap (70). Optimal cluster sizes were obtained by computing the various the threshold statistic for agglomerative clustering and finding the optimal silhouette score. Once iModulons clusters were calculated, clusters that had above average Pearson R correlation between iModulons were manually inspected to determine physiological function.

### Generating iModulonDB dashboards.

iModulonDB dashboards were generated using the PyModulon package (14, 18). Where applicable, we provide links to gene information in Mycobrowser (78).

10.1128/msphere.00033-22.5 DATA SET S5 iModulons that were found to be differentially regulated across different phases during the hypoxia time course. Download Data Set S5, XLSX file, 0.01 MB. Copyright © 2022 Yoo et al. 2022 Yoo et al. https://creativecommons.org/licenses/by/4.0/ This content is distributed under the terms of the Creative Commons Attribution 4.0 International license.

10.1128/msphere.00033-22.6 DATA SET S6 iModulons that were found to be differentially regulated across various M. tuberculosis infection events. Download Data Set S6, XLSX file, 0.01 MB. Copyright © 2022 Yoo et al. 2022 Yoo et al. https://creativecommons.org/licenses/by/4.0/ This content is distributed under the terms of the Creative Commons Attribution 4.0 International license.
